# Genetic variation and relationship among content of vitamins, pigments, and sugars in baby leaf lettuce

**DOI:** 10.1002/fsn3.1196

**Published:** 2019-08-30

**Authors:** Ivan Simko

**Affiliations:** ^1^ U.S. Department of Agriculture Agricultural Research Service U.S. Agricultural Research Station Crop Improvement and Protection Research Unit Salinas CA USA

**Keywords:** baby leaf lettuce, genetic variation, human health, pigments, sugars, vitamins

## Abstract

Baby leaf lettuce harvested approximately 30 days after planting is the primary component of spring mix salads, a popular type of packaged salads. Very little is known, however, about the content of vitamins, sugars, and pigments in young lettuce plants. Therefore, plants of 42 accessions harvested at baby leaf stage were analyzed for the contents of vitamin C, ß‐carotene, anthocyanins, chlorophylls, glucose, fructose, and sucrose. Significant differences among accessions were found for content of all seven compounds plus sucrose sweetness equivalency (*SSE*) and average vitamin load (AVL_AC_). “Floricos” was highest in all sugars, *SSE* and vitamin C; “Taiwan” was highest in ß‐carotene and AVL_AC_, and “Annapolis” and “Darkland” were highest for anthocyanins and chlorophyll contents, respectively. The lowest content of glucose and sucrose was found in iceberg “Salinas,” fructose in *L. serriola* accession UC96US23, vitamin C in PI 257288, and β‐carotene in “Solar.” The lowest relative sweetness (*SSE*) was calculated for UC96US23, followed by “Salinas,” while the lowest AVL_AC_ was estimated for PI 257288. There were very strong, positive correlations among contents of the three sugars, and between β‐carotene and vitamin C, and β‐carotene and anthocyanins. Composition profiles of accessions presented in this study, together with identified associations between compounds, can be used by breeders, growers, and producers to select lettuces with desirable combinations of sugars, pigments, and vitamins. This information can help in development of new cultivars and breeding lines with desirable combination of traits, pleasing taste, and higher vitamin content.

## 1 INTRODUCTION

Lettuce (*Lactuca sativa* L.) is the most popular, commercially grown leafy vegetable in many countries around the world (Simko, Hayes, Mou, & McCreight, 2014). Lettuce leaves, which are mainly consumed raw, contain dietary fiber, several important dietary minerals, vitamins (e.g., vitamin B9 and vitamin C), and bioactive compounds (e.g., carotenoids and phenolic compounds; Kim, Moon, Tou, Mou, & Waterland, 2016; Mou, 2009) that contribute to human nutritional benefits. Baby leaf lettuce is the primary component of spring mix salads that are popular type in packaged salads. Lettuce for baby leaf production is harvested when the first four true leaves reach the length of circa 5–13 cm, approximately 30 days after planting.

Plants are an important source of phytochemicals (Dillard & German, 2000) and vitamins needed for proper functioning of human organisms and prevention of vitamin‐related deficiencies, such as blindness (vitamin A), beriberi (vitamin B1), pellagra (vitamin B3), anemia (vitamin B6), scurvy (vitamin C), and rickets (vitamin D; Asensi‐Fabado & Munné‐Bosch, 2010). Vitamin C is also required for biosynthesis of collagen and certain hormones, and has a therapeutic potential in cancer and heart disease (Li & Schellhorn, 2007). Three basic types of pigments that cause coloration of lettuce leaves are mainly chlorophylls (green color), anthocyanins (red‐purple color), and carotenoids (yellow‐orange color that is usually masked in photosynthetically active tissue). Consumption of plant pigments has a beneficial effect on human health (Khoo, Azlan, Tang, & Lim, 2017). Epidemiological studies have shown positive associations between increased intake of carotenoids and decreased the risk of cancer (Tanaka, Shnimizu, & Moriwaki, 2012). Certain carotenoids, that are precursors for vitamin A biosynthesis, also have important roles in prevention of blindness due to age‐related macular degeneration (Taylor & Ramsay, 2005). Anthocyanins are prominent phenolic compounds found abundantly in red‐colored lettuce (Simko, Hayes, & Furbank, 2016; Sytar et al., 2018). Anthocyanin‐rich lettuce demonstrated antidiabetic effects and may help in improving metabolic syndrome conditions of fatty liver and glucose metabolism (Cheng et al., 2014). Chlorophylls and their derivatives showed a positive effect as a cancer preventative agent (Ferruzzi & Blakeslee, 2007). The effect was attributed to chlorophylls’ antioxidant and antimutagenic activity, mutagen trapping, modulation of xenobiotic metabolism, and induction of apoptosis (Ferruzzi & Blakeslee, 2007). Though lettuce is not a significant source of sugars (Mou, 2009) in a human diet, the presence of sugars in lettuce leaves substantially affects sensory perception of their taste (Chadwick, Gawthrop, Michelmore, Wagstaff, & Methven, 2016).

The content of bioactive compounds in lettuce is significantly influenced by a growing environment (Riga et al., 2019; Sytar et al., 2018) and the plant genotype (Mou, 2005; van Treuren, Eekelen, Wehrens, & Vos, 2018; Yang et al., 2018). The objective of the present study was to analyze the content of vitamin C, ß‐carotene, anthocyanins, chlorophylls, glucose, fructose, and sucrose in baby leaf lettuce and to investigate relationships among contents of these compounds in 42 lettuce accessions. Most of the studies related to the content of vitamins, pigments, and sugars in lettuce were previously performed on plants harvested at full maturity (approximately 60–90 days after planting). Relatively little is known, however, about the content of these compounds in baby leaf lettuce, the product that became a highly popular part of a human diet in recent years.

## 2 MATERIAL AND METHODS

### 2.1 Plant material and growth conditions

A set of 42 lettuce accessions evaluated in this study included 30 cultivars, six plant introductions, five breeding lines, and a single accession of *Lactuca serriola* L., the wild species closely related to cultivated lettuce (Table 1). Seeds were planted in potting soil (Premium Growers Mix, Sun Land Garden Products), covered with sand and wetted. Trays with seeds were kept for 48 hr at 10°C in the dark to improve uniformity of germination. Afterward, the trays were transferred for two weeks to a growth room with 20°C and 16‐hr/8‐hr light/dark photoperiod for germination and initial growth. Established, uniform‐looking plants were transplanted to 7.6 cm pots containing 1:1 mix of potting soil and sand, fertilized with ½ tbsp of Osmocote Smart‐Release Plant Food Flower & Vegetable (Scotts), and grown in a greenhouse until four true leaves on majority of plants reached circa 10 cm. Average daily temperature in the greenhouse (April, May 2018) ranged from 20 to 24°C, day length ranged from approximately 13 to 14 hr, and outdoor average daily light integrals were between 40 and 55 mol m^‐2^ day^‐1^. Plants were grown in the Randomized Complete Block design with four replications (42 × 4 plants in total). Each of the four individual plants per accession was used for quantification of compounds.

**Table 1:** List of 42 lettuce accessions tested in the present study, their classification, color, and content of sugars, vitamins, and pigments

Accession name	Accession NPGS number^a^	Lettuce horticultural type or species^b^	Color appearance	Glucose (g/kg FW)	Fructose (g/kg FW)	Sucrose (g/kg FW)	Vitamin C (mg/kg FW)	β‐carotene (mg/kg FW)	Chlorophylls (SPAD)	Anthocyanins (ACI)
Alpi	PI 657632	Romaine	Green	3.60	5.92	0.06	147	47.4	43.3	7.7
Annapolis	PI 690778	Romaine	Dark red	2.02	3.58	0.07	131	100.5 H	34.2	17.9 H
Balady Barrage	n.a.^c^	Stem	Dark green	5.27 H^d^	9.01 H	0.60	154	52.7	35.9	5.5
Balady Cairo	PI 667835	Romaine	Light green	4.29 H	6.40 H	0.38	84 L	24.4	31.8 L	4.6
Bandit	n.a.	Romaine	Green	5.76 H	9.04 H	0.72 H	120	31.7	42.0	7.6
Caesar	PI 595832	Romaine	Green	5.47 H	8.24 H	0.62	165 H	46.9	48.7	7.7
Dark Green Romaine	n.a.	Romaine	Dark green	2.54	4.90	0.19	112	46.0	45.2	8.1
Darkland	PI 539937	Romaine	Dark green	2.98	5.50	0.21	163 H	67.5	52.3 H	9.6
Eruption	PI 613577	Latin	Green–red	0.44 L	1.32 L	0.05	153	55.8	35.6	15.8 H
FLA24069	n.a.	Romaine	Dark green	1.64	4.29	0.05	175 H	39.3	43.2	8.0
Floricos	PI 617956	Romaine	Dark green	6.81 H	11.56 H	1.48 H	197 H	43.0	45.3	7.1
Gardenia	n.a.	Romaine	Green	0.41 L	1.38 L	0.05	116	44.7	36.3	6.2
Green Forest	n.a.	Romaine	Green	2.39	4.68	0.05	109	41.8	42.9	8.5
Green Towers	PI 601336	Romaine	Green	6.15 H	8.63 H	0.53	135	55.3	41.6	8.0
Heavy Heart	n.a.	Romaine	Green	0.96 L	3.24 L	0.06	72 L	41.9	46.5	7.7
Inverno De Mall	n.a.	Romaine	Green	4.22 H	7.12 H	0.25	117	25.4	39.1	5.5
La Brillante	n.a.	Batavia	Light green	0.65 L	1.70 L	0.05	103	31.3	28.2 L	3.3 L
Lee Tal	PI 665194	Romaine	Green	1.17 L	2.70 L	0.15	104	31.2	34.8	5.3
Little Gem	PI 617959	Latin	Green	1.64	3.87	0.05	64 L	36.6	39.1	6.4
Little Lepricon^e^	PI 617947	Romaine	Green–red	2.71	5.23	0.32	114	51.4	38.3	7.9
Merlot	PI 667702	Leaf	Red	0.35 L	0.87 L	0.06	78 L	31.8	29.3 L	11.8 H
Parris Island Cos	PI 665200	Romaine	Green	6.13 H	9.32 H	1.01 H	99	25.6	51.6 H	7.8
Queen of Hearts	PI 628367	Romaine	Dark green	1.86	4.34	0.12	151	39.4	47.4	8.2
Romaserra	n.a.	Romaine	Green	3.06	4.81	0.20	116	31.7	39.8	4.8
Rubicon	PI 634552	Romaine	Dark green	4.27 H	7.08 H	0.24	131	40.5	44.1	9.3
Salinas	PI 536851	Iceberg	Green	0.12 L	0.49 L	0.03	99	56.5	40.4	5.8
SalVal‐321	n.a.	Romaine	Green	1.35 L	3.60	0.08	110	36.2	46.3	7.6
Siskiyou	PI 612428	Romaine	Green	3.90	6.46 H	0.45	110	33.2	36.5	6.1
SM09B	PI 658679	Romaine	Dark green	2.68	5.25	0.24	104	55.2	47.9	9.8
SM13‐R2	W6 50790	Romaine	Green	4.62 H	7.02 H	0.20	131	54.5	45.1	8.2
SM13‐R3	W6 50791	Romaine	Dark green	3.48	6.96 H	0.07	70 L	32.0	50.0	8.0
Solar	n.a.	Romaine	Light green	2.85	6.94 H	0.89 H	136	15.2 L	31.9 L	4.2
Taiwan	n.a.	Romaine	Dark green	0.74 L	0.98 L	0.08	186 H	152.9 H	44.1	8.3
UC96US23	n.a.	*L. serriola*	Dark green	0.17 L	0.26 L	0.09	137	87.9 H	41.1	6.5
Ultegra	PI 604241	Romaine	Dark green	4.07 H	7.66 H	0.21	116	52.1	45.2	8.3
Valmaine	PI 543959	Romaine	Green	1.34 L	2.88 L	0.06	178 H	68.0	48.1	8.6
n.u.^f^	PI 257288^g^	Romaine	Dark green	2.95	5.37	0.12	61 L	28.4	39.8	6.0
n.u.	PI 278074	Romaine	Green	1.08 L	2.54 L	0.05	118	53.4	36.3	4.7
n.u.	PI 278100‐COS	Romaine	Green	1.27 L	2.76 L	0.05	105	38.8	46.3	5.9
n.u.	PI 358033‐COS^h^	Romaine	Green	1.89	3.60	0.06	133	33.8	32.4	4.1
n.u.	PI 491086	Romaine	Dark green	0.71 L	2.65 L	0.10	134	126.2 H	47.4	12.5 H
n.u.	PI 665200^i^	Romaine	Dark green	2.76	5.59	0.21	117	52.6	40.9	7.3
Overall mean				2.68	4.90	0.25	123	49.1	41.3	7.7

a Identification number in the U.S. National Plant Germplasm System (NPGS) database (https://npgsweb.ars-grin.gov/gringlobal/search.aspx?) accessed on 9 July 2019.

b Species name is provided for a single, tested accession of prickly lettuce, the wild lettuce species sexually compatible with cultivated lettuce.

c Not available. This accession is not present in NPGS database.

d H and L letters indicate values that are significantly (*p* < .05) higher (H) or lower (L) than the overall mean as determined by analysis of means (ANOM).

e Possibly the same accession as “Red Leprechaun” in NPGS.

f Name unknown. There is no name (or only unverified name) for this entry in NPGS database. Plant introduction (PI) number in the second column was used to identify this accession in the present study.

g Unverified name “Oreja.”

h Unverified name “Bela Marula.”

i Selection from “Parris Island Cos” (PI 665200) made at the USDA‐ARS station in Salinas, California. Because this selection appears to be phenotypically distinct from the original cultivar, it was tested as a separate accession.

### 2.2 Quantification of compounds in lettuce leaves

The relative content of chlorophylls and anthocyanins was determined two days before harvest using SPAD‐502 (Spectrum Technologies) and ACM‐200 plus (Opti‐Sciences) hand‐held meters, respectively. These devices use light transmittance to provide good *in situ* estimates of relative contents of the two pigments (van den Berg & Perkins, 2005; Parry, Blonquist, & Bugbee, 2014). Chlorophylls and anthocyanins were measured on three leaves of similar age (avoiding youngest and oldest leaves) and size (circa 10 cm) per plant. The measuring clip was positioned about 1 cm from the edge of the leaf while ensuring that major veins were avoided. The content of chlorophylls is expressed in SPAD units; the content of anthocyanins is expressed in ACI (anthocyanins content index) units. For each plant, the averages of three measurements of chlorophylls and anthocyanins were recorded and used in statistical analyses.

All leaves of a plant (devoid of stem tissue) were harvested, split into three homogeneous samples, and used for analyses of sugars, vitamin C, and β‐carotene. All laboratory analyses were performed by UC Davis Analytical Laboratory (https://anlab.ucdavis.edu). Samples for analyses of soluble carbohydrates were dried at 55ºC, ground, and extracted with hot deionized water (Johansen, Glitsø, & Bach Knudsen, 1996). The amounts of glucose, fructose, and sucrose in extracts were determined using PerkinElmer Series 200 Quaternary HPLC (PerkinElmer) with Sciex API 200 mass spectrometer (Sciex). HPLC was performed with Luna NH2 (250 mm × 4.6 mm, 5 µm particle size, 100 Å) column and C18 SecurityGuard guard column (4 mm × 3 mm) (both from Phenomenex). Mobile phase consisted of a filtered and degassed mixture of acetonitrile and water (78:22) run at a flow rate of 2.75 ml/min. Typical retention times were 2.45 min for fructose, 2.85 min for glucose, and 3.70 min for sucrose. Column eluates corresponding to analyzed carbohydrates were collected, diluted, and used for mass spectrometry. Mass spectrometer parameters were as follows: negative ion atmospheric‐pressure chemical ionization, multiple ions scan, duration 2.5 min, curtain gas (CUR = 50), needle current (NC = −5), temperature (TEM = 400), gas 1 (GS1 = 50), gas 2 (GS2 = 40), declustering potential (DP = −40), focusing potential (FP = −200), and entrance potential (EP = −4). The amount of three sugars was calculated using a second order internal standard curve with 1/x weighting, plotting the ratio of analyte to internal standard concentration versus the ratio of analyte peak area to internal standard peak area. Each sugar had its own internal standard prepared from CAR10‐1KT kit (MilliporeSigma). The content of glucose, fructose, and sucrose is reported in g per kg of fresh weight (g/kg FW).

Sucrose sweetness equivalency (*SSE*) was calculated by weighing the content of glucose, fructose, and sucrose by their relative sweetness. Relative sweetness is a dimensionless quantity based on a human perception relative to that perceived for the sweetness of sucrose. Thus, the relative sweetness of sucrose is 1.00, while the values for glucose and fructose are about 0.74 and 1.17, respectively (Joesten, Castellion, & Hogg, 2007).

*SSE* = glucose content (g/kg FW) × 0.74 + fructose content (g/kg FW) × 1.17 + sucrose content (g/kg FW) × 1.00

*SSE*, that was calculated from the amount of sugars in 1 kg of leaves, is expressed in g of sucrose equivalents per kg of FW (g_SE_/kg FW).

Vitamin C (ascorbic acid) was quantified in leaf tissue according to previously developed protocol (Bouzari, Holstege, & Barrett, 2015) with minor modifications. Briefly, leaf sample homogenate (6.4 g) was mixed with 13.6 ml of 2% oxalic acid (Fisher Scientific) and centrifuged at 5,724 g for 10 min at 4°C. A 1.2 ml aliquot was taken and mixed with 400 μl of 5% dithiothreitol (VWR) to convert dehydroascorbic acid to ascorbic acid. Subsequently, the sample was filtered through a 0.2 μm filter and transferred to an autosampler vial for HPLC analysis. The amount of vitamin C was determined by PerkinElmer Series 200 Quaternary HPLC with UV/Vis diode array detection at 261 nm. A Phenomenex dual Synergi Hydro RP columns (150 mm × 4.6 mm, 4 µm particle size, 80 Å) and C18 SecurityGuard guard column (4 mm × 3 mm) in series with a gradient of 0.1% formic acid in methanol (solvent A) and 0.1% formic acid in mixture of water and methanol (95:5) (solvent B) were used. The gradient program was (time in min, flow rate in ml/min, solvent in %) 0‐0.5‐100B, 7‐0.5‐100B, 0.1‐0.5‐100A, 2‐0.5‐100A, 3‐0.8‐100A, 0.1‐0.8‐100B, 8.7‐0.8‐100B, and 0.1‐0.5‐100A. Retention time of ascorbic acid standard (MilliporeSigma) was 8.2 min.

β‐carotene was quantified from a homogenized sample prepared from 10 g FW of tissue and 6 g of deionized water. A 3.2 g aliquot was mixed with 16 ml of ethyl acetate (OmniSolv purity) containing 0.05% butylated hydroxytoluene (both from MilliporeSigma) and homogenized for 30 s. Five grams of sodium sulfate (Na_2_SO_4_) was added to the mixture, and then, the mixture was shaken vigorously 20 times and allowed to settle for at least 10 min. A 8 ml aliquot was transferred into Turbovap test tube and evaporated to dryness at 50ºC with N_2_ using Zymark TurboVap LV (Biotage USA). To dissolve the residue, 0.2 ml of ethyl acetate was added to the test tube, then 1.8 ml of methanol. The sample was vortexed and filtered prior to running on PerkinElmer Series 200 Quaternary HPLC with UV/Vis diode array detection at 450 nm. The HPLC analysis used a Synergi Max RP C18 (250 mm × 4.6 mm, 4 µm particle size, 80 Å) column (Phenomenex) with C18 SecurityGuard guard column (4 mm × 3 mm) and an isocratic mobile phase of methanol:acetonitrile (90:10) pumped at 1.2 ml/min flow rate. β‐carotene standard was purchased from MilliporeSigma. Both Vitamin C and β‐carotene contents were calculated using linear external calibration curves plotting concentrations versus peak areas. The content of vitamin C and ß‐carotene is reported in mg per kg of fresh weight (mg/kg FW), with a detection limit of 1 mg/kg.

The human body converts ß‐carotene into retinol; therefore, Recommended Daily Allowance (RDA) of vitamin A by National Institutes of Health (NIH) is given as Retinol Activity Equivalents (RAE) (NIH, 2019). Though NIH recommends the conversion rate of 12:1 from ß‐carotene into retinol (NIH, 2019), it was shown that the conversion rate for green leafy vegetables is lower, ranging from 21:1 to 28:1 (Tang, 2010). Therefore, the content of ß‐carotene was divided by 25 to get an approximate conversion into vitamin A (given as RAE). RDA of vitamin A for healthy individuals over 18 years old is 900 µg for males and 700 µg for females (average of 800 µg = 0.8 mg); RDA of vitamin C is 90 mg for males and 75 mg for females (average of 82.5 mg; NIH, 2019). To calculate % RDA attained from one 1 kg of lettuce (in FW), the following formulas were used:

Vitamin A (mg/kg FW) = ß‐carotene (mg/kg FW) ÷ 25 (conversion ratio to RAE)
Vitamin A (% RDA/kg FW) = vitamin A (mg/kg FW) ÷ 0.8 mg (RDA) × 100
Vitamin C (% RDA/kg FW) = vitamin C (mg/kg FW) ÷ 82.5 mg (RDA) × 100
Average vitamin load (AVL_AC_) of accessions (% RDA/kg FW) = (vitamin A (% RDA/kg FW) + vitamin C (% RDA/kg FW)) ÷ 2

AVL_AC_ value thus combines % RDA of vitamin A and vitamin C obtained from 1 kg FW of lettuce. This value indicates the average % RDA of the two vitamins coming from 1 kg FW of an accessions, but it does not take into consideration the balance between vitamins.

### 2.3 Statistical analysis

The content of each compound in every accession was subjected to analysis of means (ANOM) to identify accessions with quantities significantly different from the overall mean. Hierarchical clustering of accessions was performed using standardized values of each compound and Ward's minimum variance method. Principal component analysis (PCA) on compounds was done using the correlation matrix as an input data set. Two types of correlation analyses were calculated between the content of seven compounds in 42 accessions: Pearson's product–moment correlation (*r*) and Spearman's rank‐order correlation (*ρ*). To plot radar charts, the amount of each compound was transformed to the 0–100 scale. The scaled value for an accession A (A_0–100_) was calculated as A_0–100_ = (µ_A_ − µ_Min_)/(µ_Max_ − µ_Min_) × 100, where µ_A_ is the average amount of the compound detected in the accession A, µ_Min_ is the minimum average amount, and µ_Max_ is the maximum average amount of the compound found in the set of 42 accessions. All statistical analyses were performed using JMP software v. 11.1.1 (SAS Institute) and Microsoft Excel for Mac v. 16.16.5 (Microsoft).

## 3 RESULTS AND DISCUSSION

The content of glucose ranged from 0.1 g/kg FW (“Salinas”) to 6.8 g/kg FW (“Floricos”) with µ = 2.7 g/kg FW, fructose from 0.3 g/kg FW (UC96US23) to 11.6 g/kg FW (“Floricos”) with µ = 4.9 g/kg FW, sucrose from <0.1 g/kg FW (“Salinas”) to 1.5 g/kg FW (“Floricos”) with µ = 0.3 g/kg FW (Table 1, Figure S1), vitamin C from 61 mg/kg FW (PI 257288) to 197 mg/kg FW (“Floricos”) with µ = 123 mg/kg FW, β‐carotene from 15 mg/kg FW (“Solar”) to 153 mg/kg FW (“Taiwan”) with µ = 49 mg/kg FW, chlorophylls from 28 SPAD (“La Brillante”) to 52 SPAD (“Darkland”) with µ = 41 SPAD, and anthocyanins from 3.3 ACI (“La Brillante”) to 17.9 ACI (“Annapolis”) with µ = 7.7 ACI (Table 1, Figure S2). These values are similar to those previously reported for the content of chlorophylls (Xu & Mou, 2015), anthocyanins (Mamo et al., 2019) (Simko I, 2019, unpublished data), vitamin C (Albrecht, 1993; Mampholo, Maboko, Soundy, & Sivakumar, 2016), β‐carotene (Cassetari et al., 2015; Mampholo et al., 2016; Mou, 2005), glucose, fructose, and sucrose (Smoleń et al., 2015) in young or mature lettuce plants. The largest content of all three sugars was detected in romaine type “Floricos,” while the lowest contents of sugars were found in iceberg type “Salinas” (glucose and sucrose) and the *L. serriola* accession UC96US23 (fructose). These results could not be compared with earlier published data because accessions tested in those studies were different. However, it may be expected that wild species of lettuce contains less sugars than cultivated lettuce that was selected to be pleasing to the palate. Six accessions had the content of vitamin C significantly (*p* < .05) higher than the overall mean (“Floricos,” “Taiwan,” “Valmaine,” “Caesar,” “Darkland,” and breeding lines FLA24069), while four accessions had significantly higher the content of β‐carotene (“Taiwan,” “Annapolis,” PI 491086, and UC96US23). *L. serriola* (though different accessions) was previously identified as having a higher β‐carotene content than cultivated lettuce (Mou, 2005), while a small number of *L. serriola*, *L. saligna*, and *L. dregeana* accessions together with a few oilseed and stem type accessions were previously reported to contain over 500 mg of vitamin C per/kg FW (van Treuren et al., 2018).

**Figure 1:**
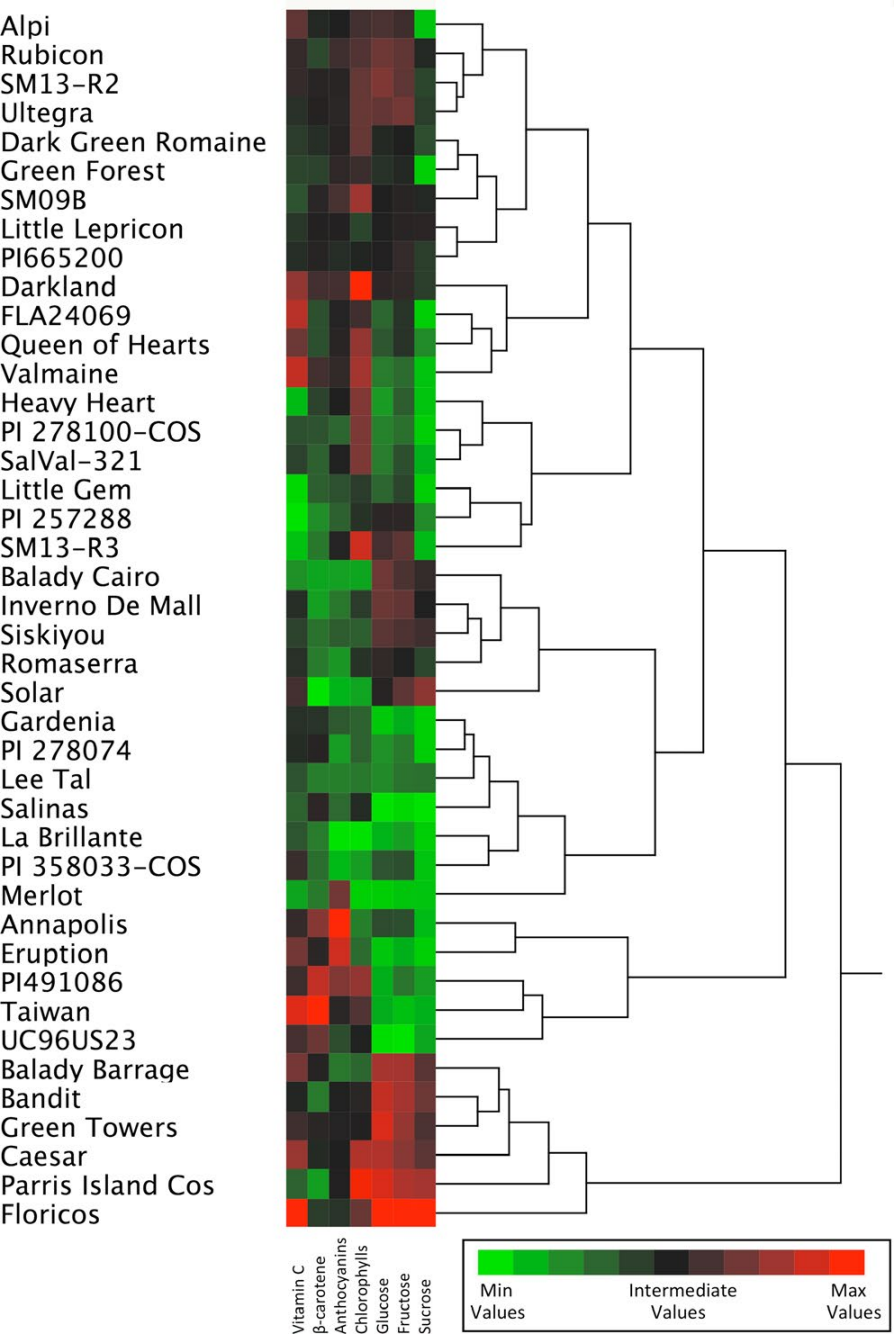
Hierarchical clustering of 42 accessions performed on standardized values of their content of glucose, fructose, sucrose, vitamin C, ß‐carotene, chlorophylls, and anthocyanins. Clustering was done using Ward's minimum variance method. The content for each compound is shown on the green–black–red scale, with green color indicating the minimal value and red color indicating the maximal value. Actual values for each compound and accession are shown in Table 1

Hierarchical clustering indicated distinct grouping of accessions with comparable contents of seven analyzed compounds (Figure 1), for example, separating the group of accessions with high content of sugars, intermediate content of pigments, and intermediate to high contentment of vitamins (“Floricos”‐like group). There was no obvious clustering of closely related accessions with known similarities in their pedigrees. For example, “Green Towers,” “Darkland,” and PI 665200 were all selected through single plant selection from “Parris Island Cos,” while “Green Towers” grouped closely to “Parris Island Cos,” “Darkland,” and PI 665200 did not group with their progenitor. In contrary, accessions without any known pedigree relationship, such as “Dark Green Romaine” and “Green Forest,” or “La Brillante” and PI 358033‐COS, or “Little Lepricon” and PI 665200, were grouped closely together. This may be due to the fact that certain compounds, for example, anthocyanins (Gurdon et al., 2019; Zhang et al., 2017) or chlorophylls (Damerum et al., 2015; Hayashi et al., 2012; Simko et al., 2016), may be produced and reach similar levels in different genotypes through involvement of different genes (pathways).

**Figure 2:**
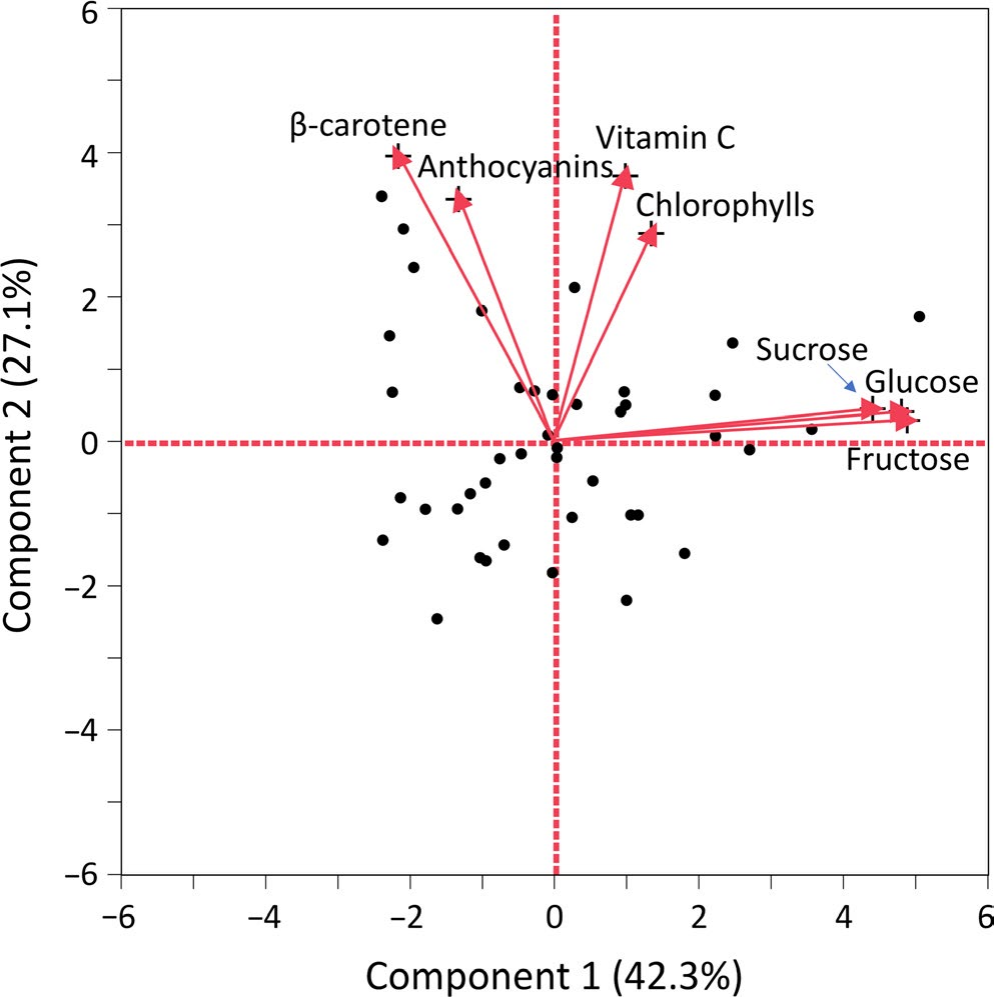
Principal component analysis (PCA) performed on 42 accessions using their content of glucose, fructose, sucrose, vitamin C, ß‐carotene, chlorophylls, and anthocyanins

Correlations that were significant using both Pearson and Spearman's correlation coefficients imply strong linear relationship among contents of three sugars (glucose, fructose, and sucrose, *r* = 0.78–0.97, *ρ* = 0.80–0.97), β‐carotene with vitamin C (*r* = 0.45, *ρ* = 0.51), and β‐carotene with anthocyanins (*r* = 0.48, *ρ* = 0.55) (Table 2). Additional correlations were identified only through Pearson's correlation coefficient (β‐carotene with both glucose and fructose, *r* = −.30 and *r* = −.36, respectively) or Spearman's correlation coefficient (chlorophylls with anthocyanins, *ρ* = 0.49). Previously, very strong, positive correlation was reported between the contents of chlorophylls and β‐carotene in mature lettuces (*r* = .82–.98) (Cassetari et al., 2015; Mou, 2005). Current study shows only a weak, nonsignificant correlation between the contents of these two compounds in baby leaf lettuce (*r* = .22, *p* = .157; *ρ* = 0.29, *p* = .065). This dissimilarity could be caused by different growing conditions, tested accessions, age of plants, analytical approaches, or a combination of multiple factors. PCA analysis confirmed very tight association among the contents of three sugars and also indicated a relationship between the contents of β‐carotene and anthocyanins, and between vitamin C and chlorophylls (Figure 2).

**Table 2:** Linear correlation coefficients between analyzed compounds in baby leaf lettuce. Upper right part of the table (above diagonal line) shows results of the Pearson correlation coefficient; lower left part of the table (below diagonal line) shows results of the Spearman correlation coefficient

Compound	Glucose	Fructose	Sucrose	Vitamin C	β‐carotene	Chlorophylls	Anthocyanins
Glucose	–	0.97***	0.77***	0.17	−0.30*	0.26	−0.12
Fructose	0.97***	–	0.78***	0.15	−0.36*	0.29	−0.13
Sucrose	0.80***	0.80***	–	0.29	−0.24	0.11	−0.16
Vitamin C	0.15	0.12	0.20	–	0.45**	0.22	0.19
β‐carotene	−0.25	−0.28	−0.19	0.51***	–	0.22	0.48**
Chlorophylls	0.23	0.27	0.15	0.17	0.29	–	0.19
Anthocyanins	−0.04	−0.03	−0.04	0.24	0.55***	0.49**	–

Note

Asterisks indicate correlation coefficients significant at: * *p* < .05, ** *p* < .01, *** *p* < .001 for *n* = 42.

The highest *SSE* was estimated for “Floricos” (20.0 g_SE_/kg FW) that has the highest contents of all three sugars (Table S1, Figure S3), while the lowest *SSE* (0.5 g_SE_/kg FW) was estimated for the *L. serriola* accession UC96US23 with very low contents of sugars. Though the relative sweetness of fructose (1.17) is higher than those of sucrose (1.00), or glucose (0.74) (Joesten et al., 2007), glucose was found to be the sugar whose content was most highly correlated with the perception of lettuce sweetness (Chadwick et al., 2016). That study found, however, that the liking of lettuce taste was not determined solely by the content of sugars, but the ratio between sweet (sugars) and bitter (sesquiterpenoid lactone) compounds. High content of sugars was pleasing for the palate, while high content of sesquiterpenoid lactone compounds (such as 8‐deoxylactucin‐15‐sulfate) was undesirable (Chadwick et al., 2016). Because neither the tasting evaluations nor the analyses of compounds related to bitter taste were performed in the present study, it is not possible to determine whether the cultivar with the highest *SSE* (“Floricos”) was also the most liked. It is expected, however, that UC96US23 taste would be generally disliked, as this wild species was the least sweet (estimate based on *SSE*) and also contains the bitter‐tasting compounds (Chadwick et al., 2016).

**Figure 3:**
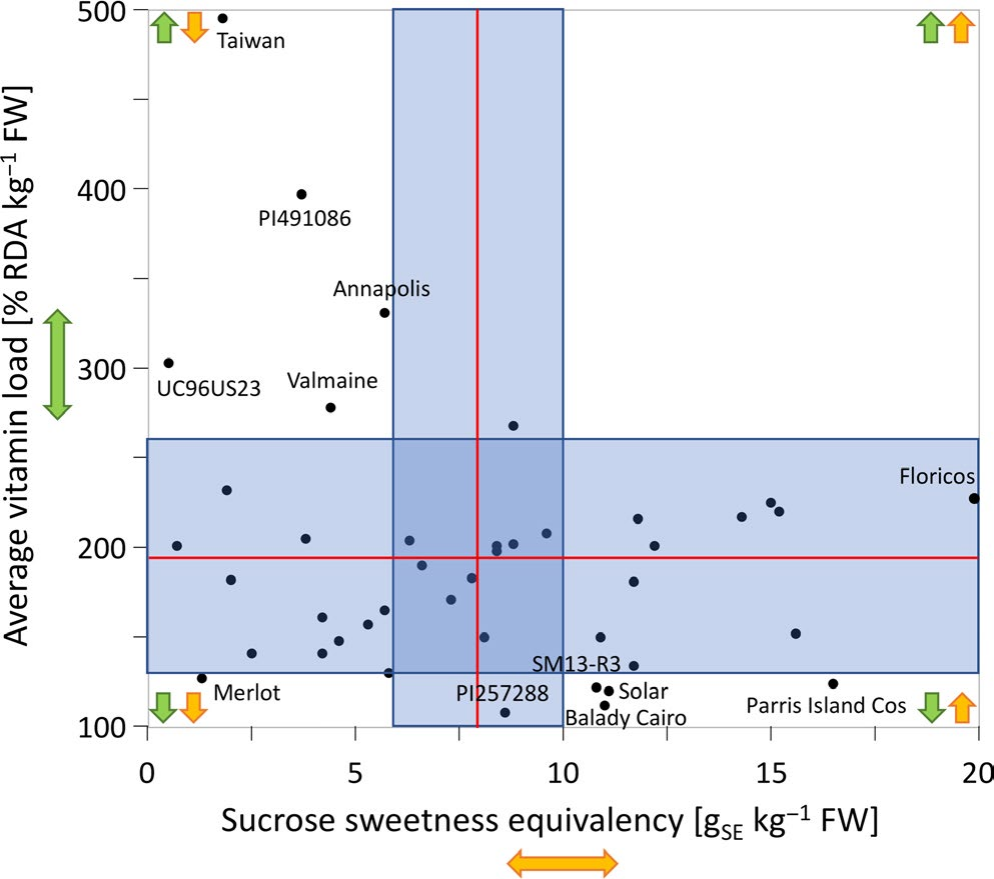
Differences in sucrose sweetness equivalency (*SSE*) and average vitamin load (AVL_AC_) among 42 lettuce accessions harvested at baby leaf stage. Analysis of means (ANOM) was performed to identify accessions with quantities significantly different from the overall mean. Values that are outside of light blue areas are significantly different (*p* < .05) from the overall means that are indicated as horizontal and vertical red lines. *SSE* was calculated from the content of glucose, fructose, and sucrose per kg of FW and multiplied by relative sweetness of sugars. AVL_AC_ value indicates percent of Recommended Daily Allowance (RDA) of vitamin A and vitamin C that is obtained from 1 kg FW of lettuce. Detailed information regarding calculations of *SSE* and AVL_AC_ is provided in material and methods. Yellow‐ and green‐colored arrows mark quadrants with high and low relative sweetness and AVL_AC_, respectively. For clarity of the figure, names are shown only for the accessions whose values are significantly different from the overall mean at both traits or have the lowest or the highest value of the trait

AVL_AC_ ranged from 108% RDA/kg FW (PI 257288) to 495% RDA/kg FW (“Taiwan”). The high AVL_AC_ value calculated for “Taiwan” was mostly due to the very high RAE of vitamin A (765% RDA/kg FW), though the content of vitamin C was also significantly higher (225% RDA/kg FW) than the overall mean (149% RDA/kg FW). For plant breeders, growers, and producers, it is important to know about both the nutritional value (e.g., AVL_AC_) and the taste attributes (such as *SSE*) of accessions. When significant differences (as compared to the overall mean) in *SSE* and AVL_AC_ were used for grouping of accessions, four of them (“Parris Island Cos,” “Balady Cairo,” “Solar,” and breeding line SM13‐R3) had high *SSE* and low AVL_AC_, five of them had low *SSE* and high AVL_AC_ (“Taiwan,” “Annapolis,” “Valmaine,” accessions PI 491086, and UC96US23), and one of them had both low *SSE* and AVL_AC_ (“Merlot”) (Figure 3, Table S1). None of the accessions was classified as having both high *SSE* and AVL_AC_, though “Floricos” seems to be closest to this group.

In addition to taste and nutritional quality, visual perception of leaf color is another important factor affecting consumers preferences. The color of lettuce leaves is predominantly determined by the amount and the ratio of chlorophylls and anthocyanins (Simko I, 2019, unpublished results). Red‐colored lettuces, such as “Annapolis,” “Eruption,” and “Merlot,” contained the highest amounts of anthocyanins (Figures S2 and S3). These cultivars likely also had significantly higher contents of flavonoids and phenolic compounds than green‐colored lettuces (Sytar et al., 2018), though such analyses were not performed at this time. Significantly high (as compared to the overall mean) levels of chlorophylls were found in two very dark green cultivars (“Darkland” and “Parris Island Cos”) (Figures [Link], [Link], [Link]). Opposite, very light green‐colored “La Brillante” had very low contents of both chlorophylls and anthocyanins (Figures S2 and S3). These data confirmed a strong relationship between the content of two pigments and visual appearance of lettuce color (Gazula, Kleinhenz, Scheerens, & Ling, 2007; Simko et al., 2016). High heritability previously detected for the contents of β‐carotene, chlorophylls (Cassetari et al., 2015), and anthocyanins (Mamo et al., 2019) indicates that new cultivars and breeding lines with desirable combination of traits could be developed.

## 4 CONCLUSIONS

Results of this study show large differences in the content of sugars, vitamins, and pigments in lettuce accessions harvested at baby leaf stage. The highest content of glucose, fructose, sucrose, and vitamin C per unit of fresh weight was detected in “Floricos,” β‐carotene in “Taiwan,” and anthocyanins and chlorophylls in “Annapolis” and “Darkland,” respectively. In contrast, the lowest content of glucose and sucrose was found in iceberg “Salinas,” fructose in *L. serriola* accession UC96US23, vitamin C in dark green romaine PI 257288, and β‐carotene in light green romaine “Solar.” Very strong, positive correlations were identified among contents of the three sugars, and between β‐carotene and vitamin C, and β‐carotene and anthocyanins. Tests in additional environmental conditions are needed to identify the magnitude of genotype × environment interaction on the content of these compounds in baby leaf lettuce. Composition profiles of accessions together with associations between compounds identified in this study can be used by breeders, growers, and producers to select lettuces with desirable combinations of sugars, pigments, and vitamins. More detailed studies are needed to determine heritability of other traits (such as sugars, sesquiterpenoid lactone compounds, and vitamins) that affect the taste and the nutritional quality of baby leaf lettuce.

## CONFLICT OF INTEREST

The author has declared that there is no conflict of interest.

## ETHICAL APPROVAL

This study does not involve any human or animal testing.
