# Supplementary figures and images for: Genetic variation and relationship among content of vitamins, pigments, and sugars in baby leaf lettuce
Source: Food Sci Nutr. 2019 Aug 30;7(10):3317–26. doi: 10.1002/fsn3.1196 (PMC6804913; doi:10.1002/fsn3.1196)

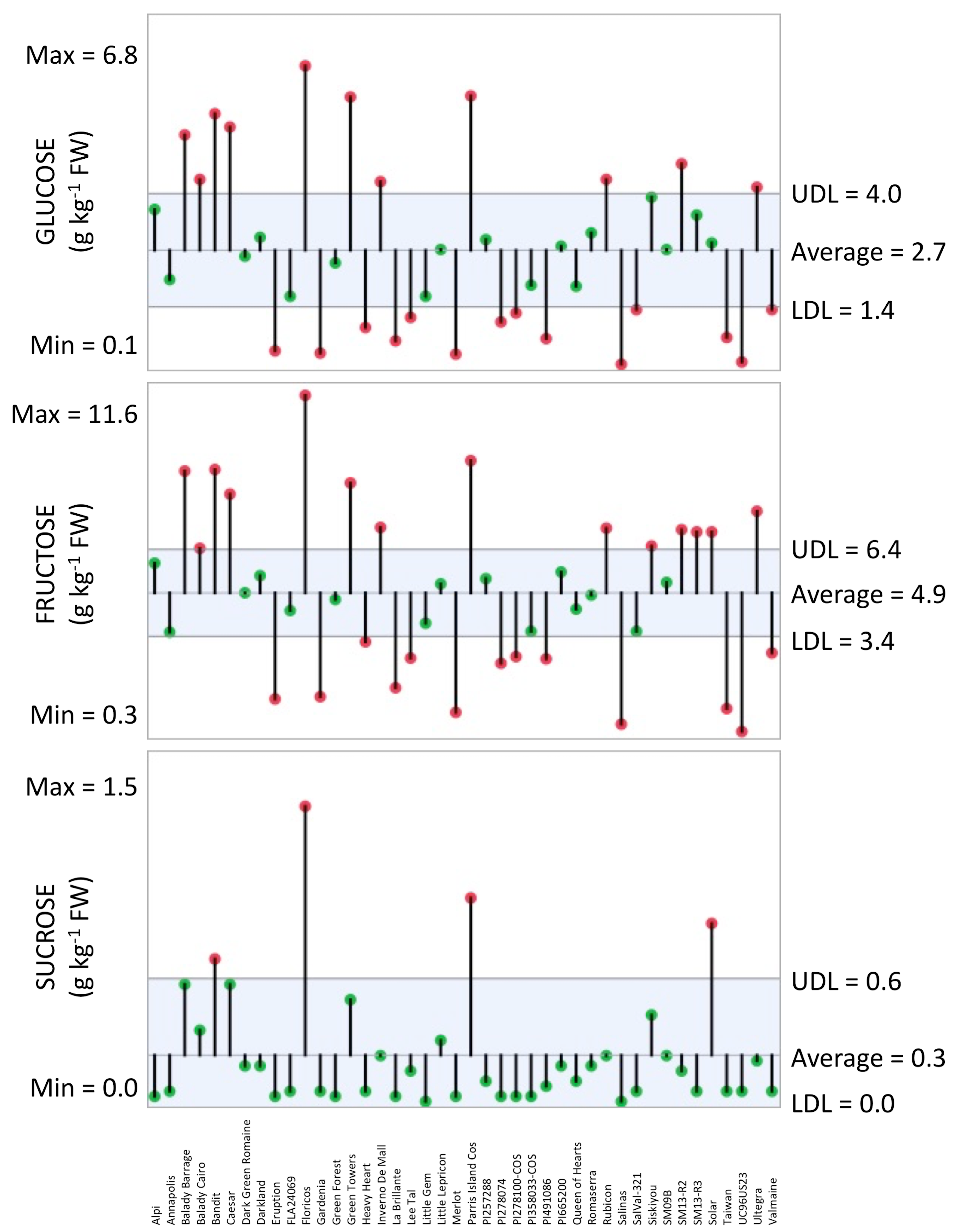

Supplement: Supplementary file 1 [file FSN3-7-3317-s001.tiff]

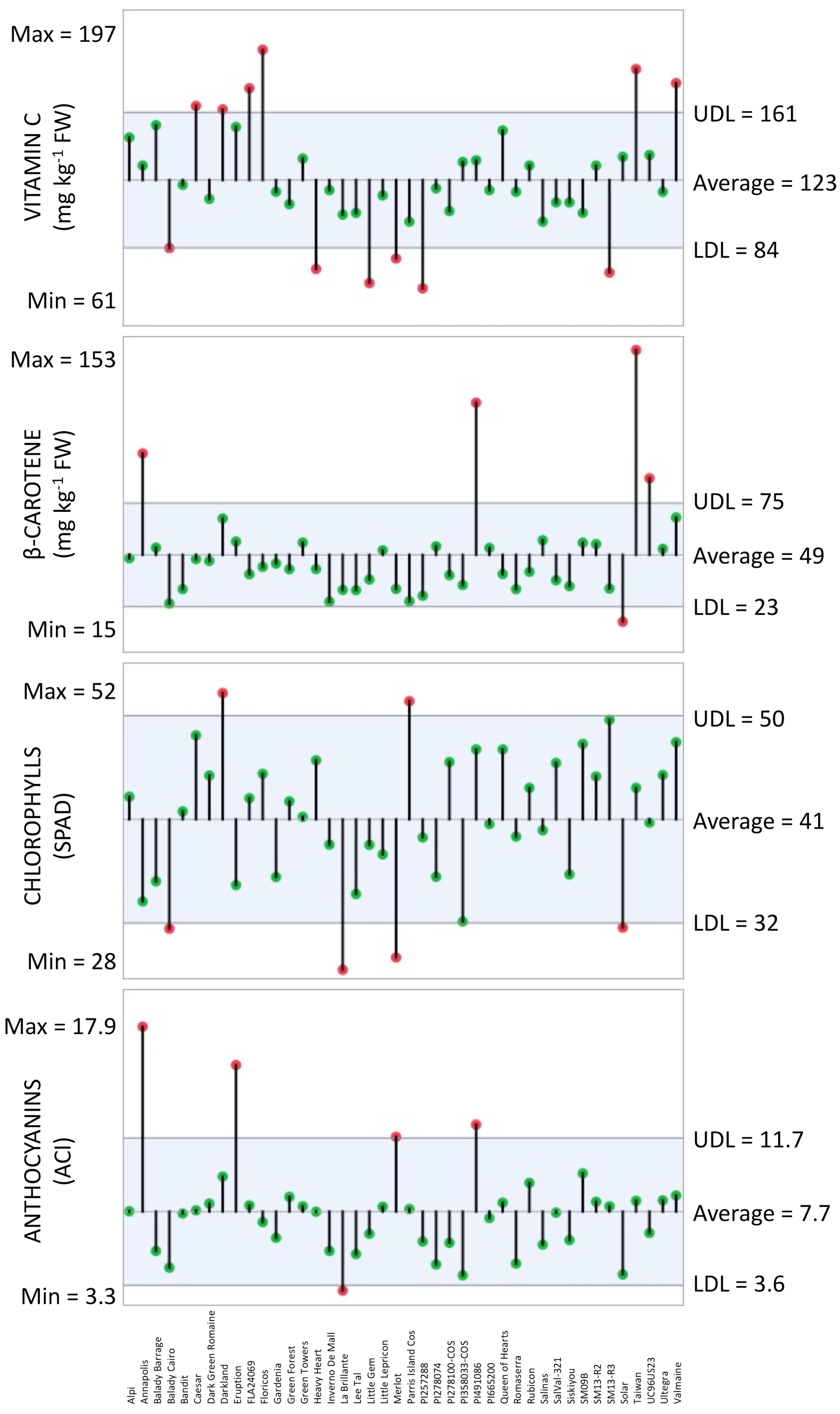

Supplement: Supplementary file 2 [file FSN3-7-3317-s002.tiff]

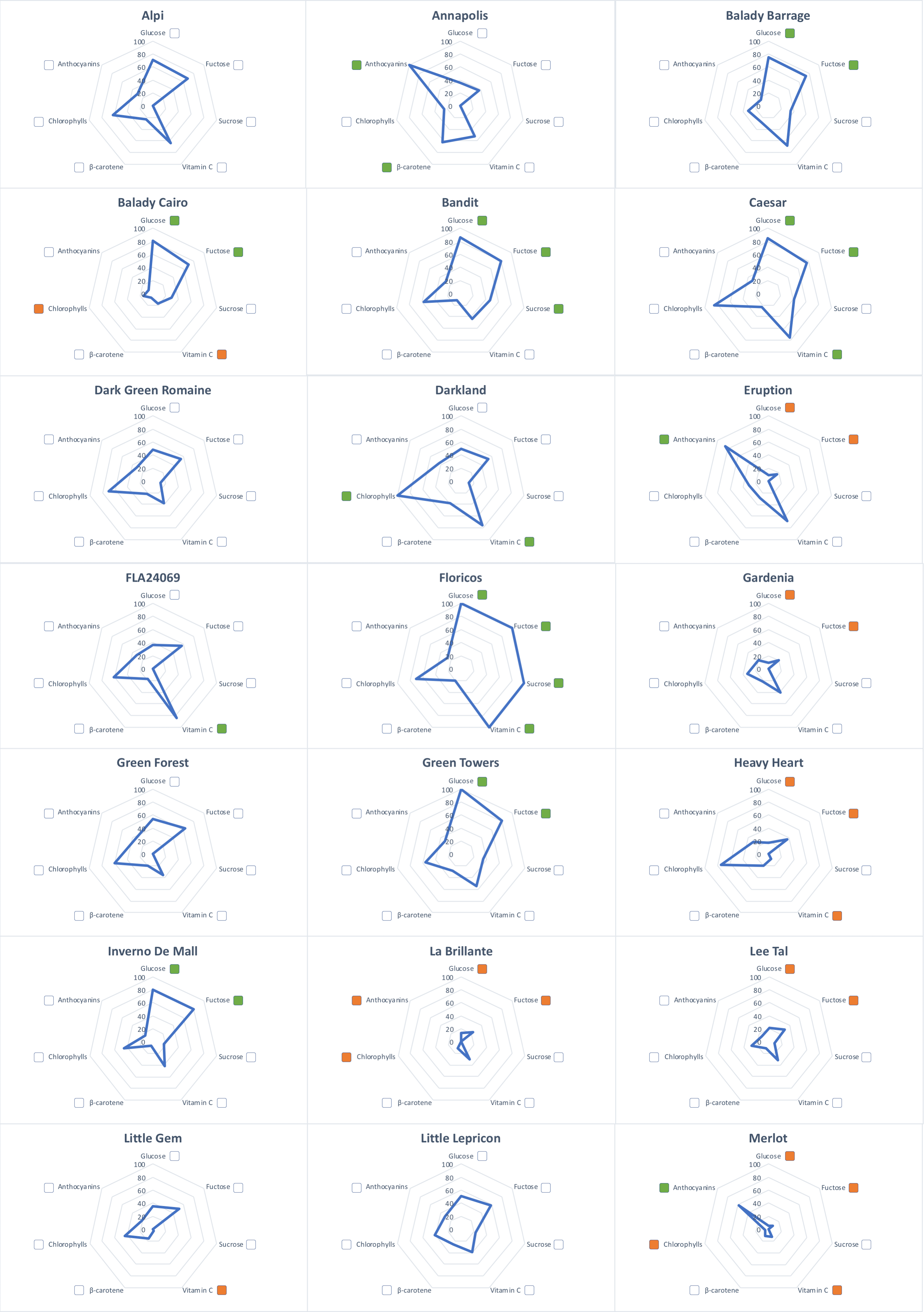

Supplement: Supplementary file 3 [file FSN3-7-3317-s003.tiff]

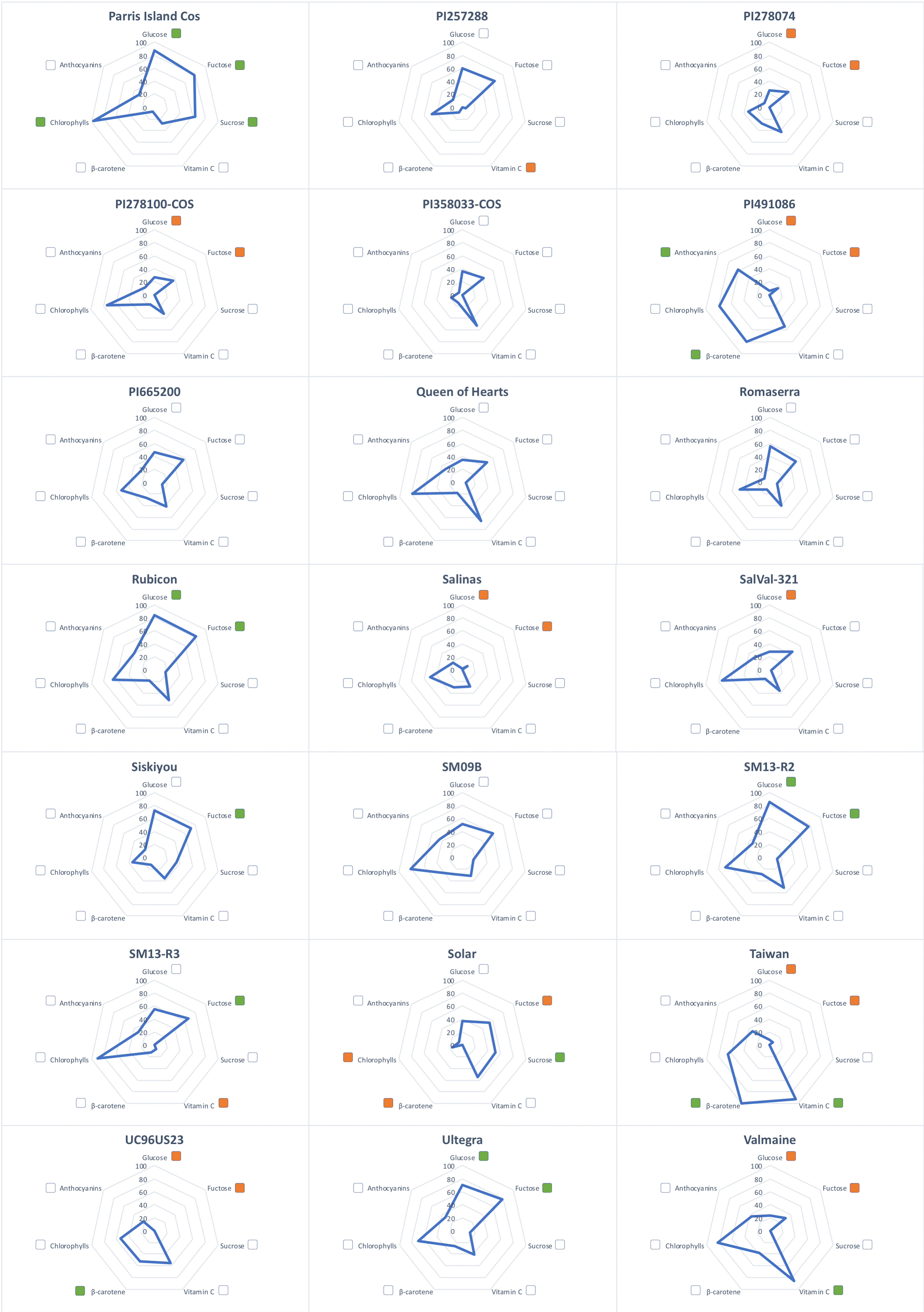

Supplement: Supplementary file 4 [file FSN3-7-3317-s004.tiff]
